# KDTViT knowledge distillation, transfer learning and transformer based deep learning framework for efficient histopathology image classification

**DOI:** 10.1038/s41598-026-49792-6

**Published:** 2026-05-05

**Authors:** Aarv Mankodi, Praveen Kumar Shukla, Hitesh Tekchandani

**Affiliations:** 1 Department of IOT & IS, Manipal University Jaipur, Jaipur, Rajasthan 303007 India; 2 Department of Artificial Intelligence and Data Science, Koneru Lakshmaiah Education Foundation, Hyderabad, Telangana 500075 India

**Keywords:** Breast cancer, Transfer learning, Knowledge distillation, Transformers, Breast cancer model, Cancer detection rate, Cancer, Computational biology and bioinformatics, Engineering, Health care, Mathematics and computing

## Abstract

Manual detection of breast cancer in histopathology images is a highly complex task due to variations in tissue appearance and the requirement for analysis across multiple magnifications levels. The often overlooked problem in most existing approaches is the number of models required for different magnification levels. Most works in this field use separate models for each magnification, which can limit scalability and practical real-world deployment. This proposed study leverages Transfer Learning and Knowledge Distillation with transformers to develop a unified model capable of handling histopathology images across multiple magnifications. The model is trained sequentially across magnification levels, enabling the transfer and refinement of learned representations. The proposed model effectively generalizes across magnifications by prioritizing common features, achieving an average accuracy of 95.43%, average precision of 94.45%, average recall of 99.20%, average F1 score of 96.76% and AUC of 0.9930 across all magnifications of the BreakHis dataset.

## Introduction

Histopathology image analysis plays a critical role in breast cancer detection. It involves microscopic examination of tissue samples to detect signs of cancer. A challenge that is faced by this analysis is the variability of tissue appearance across magnifications. Such variations can majorly affect the visual characteristics of the tissues, which has a significant impact on the classification of these tissues as cancerous or non-cancerous, making accurate classification a complex task.

Over the years, cancer has come to be known as one of the deadliest diseases in the medical field. While its detection rate has gone up in recent years, it can still have a massive impact not only on the physical health of a patient but also on the mental health and quality of life of the patient and their surrounding family and friends. The most common cancer detected is Breast Cancer. It is present mainly in the inner layers of lobules^1^.

When considering the research aspect of this field, the most common and important method used is the classification of images into various subsets. These types of works can have large variations, such as the type of images being used for classification, there can be X-ray images used, histopathology images, etc. Histopathology images are microscopic images of tissue samples, often used for analysis and disease diagnosis. For these types of problems, where the classification of images is of high importance, deep learning models such as Convolutional Neural Network models (CNNs) and transformer models are commonly used. These are deep learning models that have been tailor-made to be used for the classification of images. While these models can be applied to datasets that do not consist of images, such as tabular datasets or even signals, it is generally advised to use these models with images, as that is where these types of models can best be utilized.

It is important to note that the novelty of this work does not arise from proposing a new backbone architecture. Instead, the key contribution lies in a progressive cross-magnification training strategy that combines transfer learning with knowledge distillation to enable a single model to adapt across multiple magnification levels. Unlike conventional approaches that train separate models, the proposed framework treats magnification adaptation as a sequential learning problem, allowing efficient parameter reuse while maintaining performance across scales.

## Related work

One of the most popular datasets for breast cancer-related research is the BreakHis dataset^2^, which is a histopathology image dataset. It contains images that are classified as benign or malignant. There has been a lot of work that has been done in this field. Here, we outline prior work on this dataset using various models, along with their approaches. One approach was by Abhinav Kumar et al.^3^. Their implementation focused on using MobileHisNet on this dataset, enabling deployment on more edge devices, particularly in terms of memory usage and computational power. However, this efficiency gain is sometimes achieved at the cost of reduced representational capacity, especially when dealing with complex multi-scale variations.

Early approaches on BreakHis primarily relied on convolutional neural networks such as AlexNet, VGG, and ResNet variants^4–6^. These methods demonstrated strong local texture modeling and benefited from transfer learning. However, CNN-based architectures often struggle to capture long-range contextual relationships in histopathology images and typically require separate training pipelines for different magnification levels, increasing deployment complexity.

Recent studies have introduced transformer-based models and hybrid architectures to better capture global dependencies in histopathology images. Examples include fuzzy ViT^7^, and resolution-adaptive networks^8^. In addition, Swin Transformer-based architectures such as ST-Double Net^9^ have gained attention due to their hierarchical feature representation and improved scalability, demonstrating strong performance in medical image analysis tasks. Hybrid CNN–Transformer models, which combine local feature extraction with global context modeling, have also been widely explored to enhance classification performance. While these models improve representation learning and achieve competitive results, they often introduce higher computational costs and increased model complexity, making real-world deployment challenging. Furthermore, most of these approaches are still designed for single-magnification settings or require separate training pipelines for different magnifications, limiting their applicability in multi-scale histopathology analysis.

Several works focus on training strategies rather than architectural redesign, including contrastive learning, knowledge distillation, and federated learning^6,10^. While these methods improve generalization and robustness, they frequently operate within magnification-specific pipelines, requiring multiple independently trained models for practical usage. We use the implementation of knowledge distillation, complemented with transfer learning, to create a magnification-independent model that does not suffer from this issue.

Although knowledge distillation has been previously explored for histopathology image analysis, most existing approaches employ conventional teacher–student settings where a larger network transfers knowledge to a smaller model trained on the same data distribution. In contrast, the proposed KDT-ViT introduces a progressive cross-magnification distillation strategy. Instead of distilling from a larger architecture, knowledge is transferred sequentially across magnification levels (e.g., 40× → 100× → 200× → 400×). This allows the model to progressively inherit morphological representations learned at lower magnifications while adapting to finer structural details at higher magnifications. Consequently, distillation in KDT-ViT serves not only as model compression but also as a mechanism for magnification-aware knowledge transfer.

Studies on the BACH dataset similarly employ transfer learning and collaborative transformer-based approaches^11,12^, achieving strong classification performance. However, these methods typically address either binary or multiclass classification independently and do not explicitly tackle cross-magnification generalization.

Across both BreakHis and BACH studies, three consistent trends emerge: (1) architectural improvements increase accuracy but often at the cost of higher computational requirements, (2) advanced training strategies improve representation learning yet remain tied to magnification-specific models, for instance, several CNN-based and transformer-based models report results on individual magnification levels or require separate training pipelines for each scale, thereby increasing computational cost and maintenance complexity. Even recent transformer-based methods such as^8^ do not explicitly address cross-magnification generalization. And finally, (3) most approaches prioritize accuracy while overlooking unified deployment across magnifications. Consequently, existing solutions commonly produce multiple specialized models, which limit scalability and clinical usability. There exists a clear research gap in developing a unified framework capable of learning robust representations across multiple magnifications within a single model. Addressing this limitation is critical for practical clinical deployment, where images may be acquired at varying magnifications. The proposed KDT-ViT framework addresses this gap by leveraging transfer learning and progressive knowledge distillation to enable a single lightweight model capable of handling multiple magnification levels.

While knowledge distillation and multi-scale learning have been explored in prior histopathology studies, most existing approaches either train separate models for each magnification or rely on parallel multi-branch architectures that process multiple scales simultaneously. In contrast, the proposed framework introduces a progressive cross-magnification knowledge transfer strategy, where a single lightweight Mobile-ViT model is sequentially adapted across magnification levels using transfer learning and teacher–student distillation. This design enables unified magnification handling without requiring multiple specialized models or computationally heavy multi-scale pipelines, representing a key distinction from existing KD-based frameworks.

### Contribution

- A hybrid MobileViT-based framework is proposed for cross-magnification histopathology classification, enabling a single model to operate across multiple magnification levels.
- The proposed framework combines convolutional feature extraction with transformer-based global context modeling, achieving strong performance while maintaining a lightweight architecture suitable for efficient deployment.
- A progressive transfer learning strategy, along with a teacher–student knowledge distillation mechanism, is introduced to improve adaptation across magnifications while reducing catastrophic forgetting and enhancing feature transfer and improving classification robustness.
- A progressive cross-magnification knowledge distillation strategy is introduced, differentiating the proposed framework from conventional KD methods that operate within a single scale or multi-branch architectures.

## Methodology

**Fig. 1:**
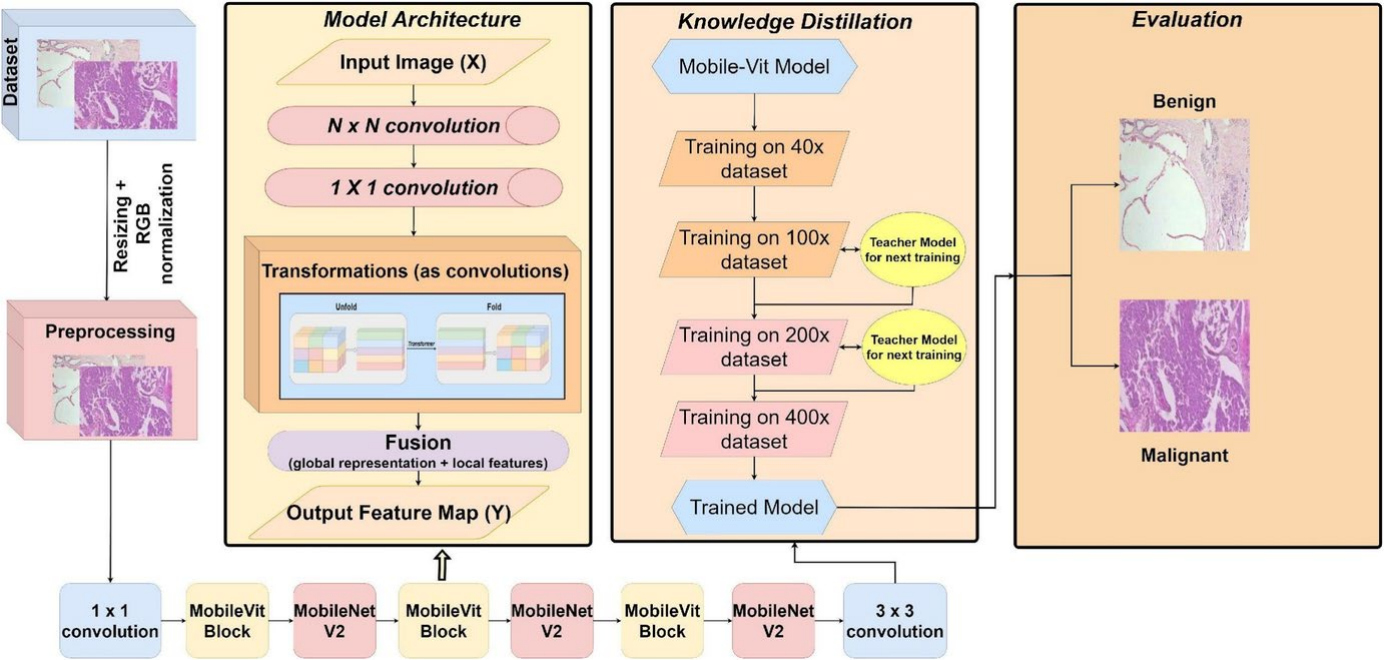
Proposed KDT-ViT architectural details.

The model that has been used for this research is a hybrid Vision Transformer model. This research proposes the architecture integrating the Mobile ViT model^13^ with knowledge distillation^14^ as depicted in Fig. 1. The methodology for this research involves various steps that are performed on the selected dataset. The pseudo code for the same has been given below with the architectural diagram.

**Algorithm 1:**
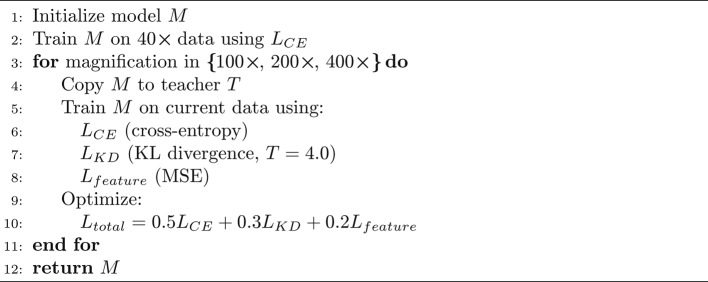
Progressive Multi-Magnification Training with KD

### Details of the proposed model architecture

Overall Architecture The model architecture proposed in this paper consists of a hybrid Vision Transformer framework based on the Mobile ViT model^13^. The architecture, as detailed in Fig. 1, combines the strengths of both convolutional neural network models, widely popular for image classification, especially in the medical field, and transformer-based models. The architecture achieves a balance and optimal tradeoff using the strengths of the CNNs and transformers to achieve greater amounts of accuracy compared to state-of-the-art models while being efficient and lightweight, and also allowing the model to extract both local and global features of an image independently, with the use of Mobile ViT as well as MobileNet blocks that alternate.

#### Architecture hyperparameters

The architectural hyperparameters used in the proposed model follow the default configuration of the MobileVit-XS configuration. The model consists of three MobileVit blocks, each incorporating transformer layers for global feature modeling.

The embedding dimensions for the MobileViT blocks are set to *d* = 96*,* 120*,* 144 for successive blocks. Each transformer block uses *L* = 2 transformer layers and *h* = 4 attention heads.

The patch size used for unfolding is *P* × *P* = 2 × 2. The feed-forward network (FFN) follows an expansion ratio of 2 the embedding dimension.

Additional architectural components include the use of depthwise separable convolutions in MobileNetV2 blocks, 1 × 1 point-wise convolutions for channel projection, and standard layer normalization with residual connections within transformer blocks.

These hyperparameters were selected to balance computational efficiency and representational capacity for histopathology image analysis.

#### CNN-based feature extraction (MobileNetV2 blocks)

The pre-processed histopathology images from the BreakHis or BACH datasets are fed into the Mobile ViT model. These images first undergo a 1 × 1 convolution operation acting as a lightweight projection layer to adjust channel dimensions before entering the deeper network. The core feature extractor of this model is built using MobileNet V2 blocks along with the Mobile ViT blocks. The purpose of the MobileNet V2 blocks is for the extraction of local features in an image. They achieve this with the use of depthwise separable convolutions. Using these allows for a significant reduction in the computational complexity of the convolution while simultaneously preserving the representational capacity of the block.

#### Transformer-based feature extraction (MobileVit blocks)

The Mobile ViT blocks are responsible for the extraction of the global features, or essentially modeling the global dependencies of the histopathology images. These blocks achieve this with the use of transformer-based self-attention mechanisms. The exact breakdown of all the parts that make up the mobile ViT block is given below, starting from the input image all the way to the output feature map.

Inside each MobileVit block, global feature interactions are modeled using transformer-based self-attention. Given an input feature **X** $\in$ R^*N*×*d*^ obtained from unfolded image patches, linear projections are applied to obtain query, key, and value matrices:

1 $${\mathbf{Q}} = {\mathbf{X}}W_{Q} ,\;\;{\mathbf{K}} = {\mathbf{X}}W_{K} ,\;\;{\mathbf{V}} = {\mathbf{X}}W_{V}$$

where *W*_*Q*_, *W*_*K*_, and *W*_*V*_ are learnable projection matrices. The self-attention operation is defined as:

2 $${\mathrm{Attention}}\left( {Q,K,V} \right) = {\mathrm{softmax}}\left( {\frac{{QK^{T} }}{{\sqrt {d_{k} } }}} \right)$$

where *d*_*k*_ represents the dimensionality of the key vectors.

For multi-head self-attention, the input is projected into multiple subspaces and attention is computed independently for each head:

3 $${\mathrm{MHA}}\left( {\mathbf{X}} \right) = {\mathrm{Concat}}\left( {head_{{1}} , \ldots ,head_{h} } \right)W_{O}$$

where each head performs scaled dot-product attention.

The output of the attention module is passed through a feed-forward network (FFN):

4 $${\mathrm{FFN}}\left( {\mathbf{X}} \right) = \sigma \left( {{\mathbf{X}}W_{{1}} + b_{{1}} } \right)W_{{2}} + b_{{2}}$$

where *σ* denotes a non-linear activation function. Residual connections and layer normalization are applied following standard transformer design. Here, *N* denotes the number of tokens obtained from the unfolded feature map, and *d* represents the embedding dimension of each token. In the multi-head attention module, *h* denotes the number of attention heads and *W*_*O*_ is the learnable output projection matrix. The parameters *W*_1_*, W*_2_ and *b*_1_*, b*_2_ represent the weights and biases of the feed-forward network.

#### Patch processing and feature transformation

The input image (denoted in Fig. 1 as X) is processed initially using a feature map of N x N size. This processing is applied to the image to allow the block to learn the localized patterns of the image. The input image is given to the block in the form of an input tensor represented as *X* $\in$ R^*H*×*W*×*C*^. The output from this standardized N x N convolution layer is then passed through a 1 × 1 or point-wise convolutional layer. This input tensor, after the application of the N x N standard convolution layer and subsequently the point-wise convolutional layer, gets converted into **X**_*L*_ $\in$ R^*H*×*W*×*d*^. While the N x N convolutional layer encodes the local information from the tensor for the block, the 1 × 1 layer learns the linear combinations of the input channels and projects the tensor onto a higher-dimensional space. The tensor input is then unfolded into N non-overlapping flattened patches, each of which is linearly projected into a higher-dimensional space. This is done to ensure that the model can learn global representations of the image with a spatial inductive bias, meaning that it can leverage the ability of CNNs to recognize and learn from the local features of an image, such as the edges, textures, etc., and use that knowledge effectively to understand and learn the images in a global context as well. This means that while other ViT models often are prone to losing the spatial order of the image, Mobile ViT is able to retain both the spatial order of the image and the patch order or global features of the images.

#### Folding and feature fusion

After this, we fold the output from the tensor to obtain **X**_*F*_ $\in$ R^*H*×*W*×*d*^. This output is then projected into a lower-dimensional space using another 1 × 1 convolutional layer. After this, all of the features are finally fused using another N x N convolutional layer. This creates the output feature map (denoted in Fig. 1 as Y) from the original input image (X). This transformation allows the model to encode both the local and the global features of the image into the output feature map/tensor. There are three fundamental steps to any convolution unfolding, matrix multiplication, and folding. Mobile ViT is similar in this regard to regular convolutions; however, it differs in the sense that it replaces the core matrix multiplication (used to understand local features) with a number of transformer layers that have deeper global processing. This allows the model to have both global focus as well as local processing, which proves useful for image training. This is the reason that this block can be considered as using transformers as convolutions.

#### Patch size

In MobileVit blocks, the input feature map is unfolded into non-overlapping patches, where the patch size directly determines the token length processed by the transformer layers. Smaller patches produce longer token sequences, enabling richer global interactions at the cost of increased computational complexity and memory usage. Conversely, larger patches reduce token length and improve efficiency but may weaken fine-grained feature representation.

Given an input feature map of spatial size *H* × *W* and patch size *P* × *P*, the resulting number of tokens is:

5 $$N = \frac{H \times W}{{P^{2} }}$$

Thus, the computational cost of self-attention grows approximately quadratically with token length. In this work, the default MobileVit patch size was adopted to maintain a balanced trade-off between computational efficiency and representation quality suitable for histopathology images.

#### Model efficiency

The computational efficiency of the proposed KDTViT model was evaluated using commonly reported deployment metrics. The model contains approximately 1.93 million trainable parameters, requiring 664.34 MMac operations per forward pass. The total model size is 7.38 MB, making it suitable for lightweight deployment scenarios. Inference efficiency was further evaluated by measuring the average inference latency, which was found to be 9.13 ms per image under an input resolution of 224 × 224. Latency measurements were performed in evaluation mode using a batch size of 1 to simulate real-world inference conditions.

These results demonstrate that the proposed architecture maintains high classification performance while preserving low computational complexity, reduced memory footprint, and fast inference speed, supporting its suitability for edge or resource-constrained medical imaging applications.

### Details on transfer learning & knowledge distillation

Transfer learning is a process in machine learning where a previously trained model’s data is used as the basis for training on future data. In this research, progressive training is done on successive magnifications. Knowledge Distillation is a method in machine learning where, when training a model on a specific dataset, a different model that has already been trained on that same dataset or a similar dataset is used as a teacher model that helps the new model get trained. This technique can be very helpful when it comes to a large dataset with successive magnifications, like the BreakHis dataset. The usage of either of these techniques without the other can cause major issues for the model training. These two techniques work hand-in-hand and back each other up. If only one of the two techniques is applied, the model risks being the victim of chronic forgetting, which is a syndrome where the model forgets what it was trained on previously (in this case, previous magnifications) and only remembers the newer training.

Transfer learning as a technique allows the model to first be trained on a broad set of data. For the paper, that would be the initial 40× magnification. When the model properly learns the features of these magnifications and when the same model learns details of later magnifications, the model uses the learning of the higher magnifications as the basis of how histopathology images are structured and what the superficial features are that make up these images. Then, when the model trains on later magnifications, it can go more in-depth on the specific features and differences that are present in later magnifications, like 200× and 400×. Where transfer learning has shortcomings for this research is that, due to the model treating the earlier magnifications as generalized representations and later magnifications as more task-specific refinements, there is a risk posed of loss of features that are specific to only the earlier magnifications, like 40× and 100×. This is where knowledge distillation becomes instrumental, as it is combined with transfer learning to provide a more balanced approach to learning for the model. Combining them, the model can prioritize the general features of earlier magnifications while also keeping a relatively strong grasp on the specific features of the same magnifications. This allows the model to learn the specific features of all magnifications while also using earlier magnifications as tools to understand general features of the dataset.

The following is a breakdown of the entire training process. A base M ViT model is first trained on the 40× magnification of the chosen dataset using standard cross-entropy supervision. The same model, with its learned weights retained, is subsequently trained on the 100× magnification with a copy of the model trained on the 40× magnification acting as a teacher model during this step in the training. This model is then copied to create a new teacher model, and the original model continues to be trained as the new student model on the 200× dataset with the teacher model for knowledge distillation. The KD objective contains the following components: (1) class balanced loss, (2) logit-level distillation using KL divergence between teacher and student softened probability distributions, and (3) feature-level distillation using mean squared error (MSE) between final representations of teacher and student models. A temperature value of *T* = 4*.*0 is used. The total optimization objective is defined as

6 $$L_{{{\mathrm{total}}}} = 0.{5}L_{{{\mathrm{CE}}}} + 0.{3}L_{{{\mathrm{KD}}}} + 0.{2}L_{{{\mathrm{feature}}}}$$

where *L*_CE_ is loss, *L*_KD_ is KL divergence between teacher and student softened logits, and *L*_feature_ is feature alignment loss. This ensures that the student model learns both class-level and magnification invariant features. Finally, this step is repeated once more as the model trained on 40×, 100×, and 200× is copied to create a teacher model, and training continues on the 400× dataset under the same distillation concept. Since all labels have the same binary label space (benign vs malignant), logits remain naturally aligned across stages. This process creates a model that has a strong understanding of the fundamentals, as it is trained repeatedly using transfer learning and has knowledge of previous magnifications distilled into it from the teacher models.

### Data set details

For this research, two histopathology datasets are used. One with multiple magnifications, known as the BreakHis Dataset, and the other with only a single magnification, called the BACH dataset. These datasets are described below.

#### BreakHis dataset

**Fig. 2:**
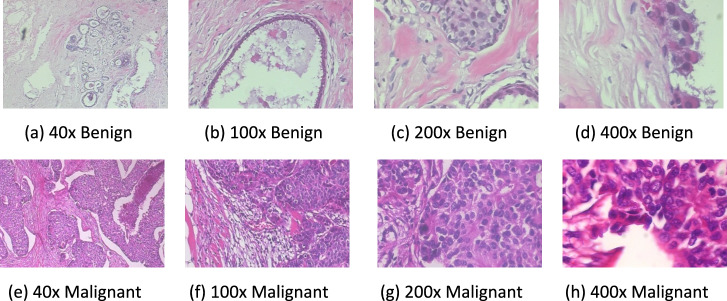
BreakHis images^2^.

One of the datasets used in this research is the Breast Cancer Histopathology Image Classification (BreakHis)^2^. The dataset, since its introduction, has been widely adopted for its large collection of labeled images. The dataset consists of a total of 7909 images, of which 2480 are classified as benign, and the other 5429 are classified as malignant. The imbalance in the dataset is addressed by using simple image augmentation, including horizontal and vertical flips and 15-degree rotations, so that the number of images in the benign class equals the number of images in the malignant class. The dataset includes four successive magnifications (40×, 100×, 200×, and 400×). Each image is in the RGB colour space and is of the size 700 × 460. Some representative images are shown in Fig. 2.

#### BACH dataset

For additional testing to be done on this model, this research has also used the BACH dataset from ICAR 2018^15^. This is another popular dataset of histopathology breast cancer images. Unlike the BreakHis dataset, which has images at multiple magnifications, the BACH dataset only contains images of 200× magnification. Along with this, the dataset also separates the images into four categories, which are in situ, invasive, benign, and normal. The benign images are of the same form as the benign images in the BreakHis dataset, and the normal images are the images where no cancer is present. In situ and invasive images are categorized, respectively, as the type of cancer that doesn’t spread from one part of the body to another and the type of cancer that does spread from one part to another.

**Fig. 3:**
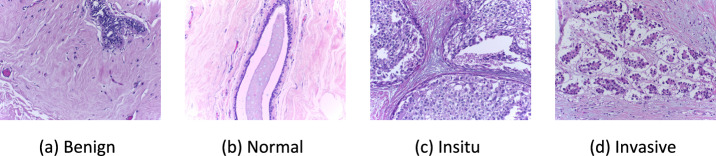
BACH images^15^.

A form of classification that can be used for this dataset is the ternary classification. Ternary classification is the type of classification where an image can be a part of one of three classes. An example of a dataset that is popular for ternary classifications is the BACH dataset^15^. Representative images of BACH dataset have been shown in Fig. 3.

#### Pre-processing of images

Pre-processing performed on the images of the dataset involves the step of normalization of the images in the Red, Green, & Blue colour space to ensure the colours of the dataset remain even and consistent. Along with this, resizing of the images from their original 700 × 460 resolution to the more widely used 224 × 224 resolution was done to fit the requirements of our desired model. This was necessary to ensure computational efficiency of the model. Although spatial down-sampling may reduce fine cellular details, the chosen resolution represents a widely adopted standard in vision transformer and CNN-based medical imaging studies, allowing consistent comparison with prior work. Furthermore, histopathology discriminative patterns such as tissue organization, glandular structure, and nuclear distribution remain visually preserved at this scale. The images are then converted into tensors to be compatible with the model’s existing framework. The dataset is split into training and test sets using tenfold cross-validation at an 80:20 ratio of training images to test images. During the training phase, the model is applied on progressive magnifications of the dataset while incorporating the implementations of knowledge distillation and transfer learning. Finally, the model is evaluated using the testing dataset.

### Training

The model is trained on all four magnifications of the BreakHis dataset. There are two main methods used to train the model; one of these is training of a singular model for all four magnifications using the aforementioned transfer learning and knowledge distillation. Another training setup used four identical Mobile ViT models, each trained on a different magnification of the dataset. This training allows for an understanding of how the Mobile ViT model is able to perform without the process of successive training on various magnifications. The training was performed using Google Colaboratory (Google Colab) software, a cloud-based notebook environment provided by Google Research^16^. The GPU used for training was the NVIDIA T4 GPU (15 GB RAM). All experiments were implemented using the PyTorch deep learning framework. The model was trained using the Adam optimizer with a learning rate of 1 10^−4^. The default Adam parameters *β*_1_ = 0*.*9, *β*_2_ = 0*.*999, and $\epsilon$ = 10^−8^ were used. Due to the lightweight nature of the model, the average training time per epoch was approximately 1–2 min, resulting in an estimated 30–60 min per fold for 30 epochs. Consequently, a full tenfold cross-validation required roughly 5–7 h per magnification level. These runtime characteristics further support the practicality of the proposed framework for research and deployment scenarios with limited computational resources. All training was performed using a tenfold cross-validation method, and each fold was run for 30 epochs per training session. GroupKFold was used to enforce slide-level separation, and class distributions were monitored across folds to ensure that benign and malignant samples remained approximately balanced. The batch size for each training was set to 32, and the learning rate was set to 0.0001. The tenfold cross-validation was implemented using GroupKFold to enforce slide-level separation. In addition, folds were constructed to maintain approximately balanced class distributions (benign and malignant) and representative samples from each magnification level to ensure stratified evaluation. Because training was performed separately for each magnification level, magnification imbalance within folds was not applicable during individual magnification experiments. For mixed-magnification experiments, folds were constructed to preserve representative samples from each magnification level. The learning rate and batch size were kept constant across all experiments to ensure consistent comparison between magnification levels and training strategies. Preliminary exploratory runs indicated that these settings provided stable optimization behavior for MobileVit-XS. Although hyperparameter sensitivity analysis may further refine performance, it was not explored in this work due to the high computational cost associated with repeated cross-validation experiments.

The BreakHis dataset exhibits class imbalance between benign and malignant samples. To mitigate bias toward the majority class, class-balanced weighting was incorporated into the loss function. Specifically, weighted cross-entropy was employed, where class weights were computed inversely proportional to class frequency. This encourages the model to assign higher importance to minority-class samples during optimization and improves robustness under imbalanced data distributions.

For training on the BACH dataset, we have used 2 methods: binary classification, where the benign images are one class, and the in-situ and invasive images are combined to form the malignant class. Since each type has 100 images, data augmentation is used for the benign images. For augmentation, only basic and widely used augmentation processes were implemented. This includes horizontal and vertical flips, along with 15-degree rotations of the images equally in clockwise and counterclockwise directions. Augmentation was applied equally to each magnification to ensure fairness in training. The other method used was ternary classification, where the three classes were benign, malignant (both the same as the binary classification above), along with the normal images as the third class. Since the Bach dataset only contains one magnification, unlike the BreakHis dataset, which has progressively increasing magnifications, both transfer learning and knowledge distillation were not used for training on the Bach dataset. For training on both datasets, we ensured patient-level separation to prevent any potential data leakage between training and testing sets. All images and slides belonging to a single patient were assigned to either the training or testing subset with no subset leakage. We have also ensured slide-level splitting to avoid any leakage between the training and testing images. The usage of slide-level splitting allows for the images from the same slide to remain in the same section, which allows the training process to avoid leakages from images of the same slide. Both of the model were trained with the use of RGB normalization on the dataset images before training. The augmentation performed for the BACH dataset was only used for training of the dataset, and testing was performed on the unaugmented dataset portion.

## Results

For the BreakHis dataset, we have trained the model such that it differentiates between the images and labels as either ’Benign’ or ’Malignant’. This classification is then used to create an accuracy percentage that, along with various other metrics, is used for evaluating the model’s performance. A similar process is used for evaluation on the BACH dataset. The four main evaluation criteria used for this research are the accuracy, precision, recall, and F1 score. Accuracy is used to measure the correctness of the predictions made by the model, precision measures the model’s ability to not make false predictions, recall or sensitivity measures the model’s ability to identify only correct instances, and the F1 score is a harmonic mean of precision and recall, giving a balance of the two.

Since the various variations of Mobile ViT models were trained separately, along with the addition of various techniques like Knowledge distillation and transfer learning, multiple iterations of training on the models have created results that are quite varying. Here, we are presenting each of these results and understanding their significance. Starting with the results obtained from training the Mobile ViT model separately on each magnification, all such parameters shown in Table 1 average around 98–99%. This means that the goal for the future training that includes multiple magnifications is to get as close to this 97% average as possible to minimize loss due to training on multiple magnifications. These numbers obtained when training at individual magnifications are then further backed up from the training done on the BACH dataset, where both the binary classification and the ternary classification result in a similar 98% accuracy.

**Table 1:** Performance Comparison on BreakHis and BACH datasets.

Model	Dataset	Mag	Acc (%)	CI	Pre (%)	Rec (%)	F1 (%)
MViT_xs	BreakHis	40×	99.01 ± 0.40	[98.51, 99.51]	99.03	99.10	98.95
MViT_xs	BreakHis	100×	98.00 ± 0.52	[97.35, 98.65]	97	99	98
MViT_xs	BreakHis	200×	99.45 ± 0.30	[98.86, 100.0]	100	99	99
MViT_xs	BreakHis	400×	97.00 ± 0.60	[96.26, 97.74]	98	98	100
MViT_xs (all magnifications combined)	BreakHis	40×	91.03 ± 0.54	[90.36, 91.70]	89	98	94
		100×	92.56 ± 0.32	[92.16, 92.96]	91	99	95
		200×	94.98 ± 0.67	[94.15, 95.81]	94	99	96
		400×	93.65 ± 0.20	[93.40, 93.90]	92	98	95
MViT_xs	BACH (binary)	200×	98.00 ± 0.5	[97.38, 98.62]	96.60	99.50	98.03
MViT_xs	BACH (ternary)	200×	98.2 ± 0.4	[97.70, 98.70]	97.20	98.86	98.34
MViT_xs + TL	BreakHis	40×	87.00 ± 0.70	[86.13, 87.87]	86	96	91
		100×	91.00 ± 0.62	[90.23, 91.77]	92	94	93
		200×	95.00 ± 0.48	[94.40, 95.60]	95	98	97
		400×	97.00 ± 0.30	[96.63, 97.37]	97	99	97
MViT_xs + KD	BreakHis	40×	80.00 ± 0.90	[78.88, 81.12]	80	93	86
		100×	87.00 ± 0.75	[86.07, 87.93]	87	95	91
		200×	93.00 ± 0.55	[92.32, 93.68]	94	97	95
		400×	96.00 ± 0.38	[95.53, 96.47]	97	99	98
MViT_xs (Freeze) + TL + KD	BreakHis	40×	85.00 ± 0.68	[83.7, 86.3]	89	89	89
		100×	85.00 ± 0.61	[83.8, 86.2]	94	85	89
		200×	86.00 ± 0.59	[85.27, 86.73]	90	90	90
		400×	89.00 ± 0.50	[88.38, 89.62]	89	96	92
MViT_xxs + TL + KD	BreakHis	40×	81.00 ± 0.80	[80.01, 81.99],	82	96	87
		100×	94.00 ± 0.60	[93.26, 94.74]	93	97	95
		200×	95.00 ± 0.35	[94.57, 95.43]	97	97	97
		400×	96.00 ± 0.46	[95.43, 96.57]	98	98	98
MViT_s + TL + KD	BreakHis	40×	81.00 ± 0.78	[80.03, 81.97]	80	96	87
		100×	94.00 ± 0.64	[93.21, 94.79]	92	97	95
		200×	96.00 ± 0.45	[95.44, 96.56]	97	98	98
		400×	98.00 ± 0.50	[97.38, 98.62]	99	99	98
(proposed) KDT-ViT [MViT_xs + TL + KD]	BreakHis	40×	92.58 ± 0.53	[91.92, 93.24]	91.56	98.25	94.79
		100×	95.10 ± 0.42	[94.58, 95.62]	93.89	99.37	96.55
		200×	96.57 ± 0.38	[96.10, 97.04]	95.46	99.78	97.57
		400×	97.47 ± 0.27	[97.13, 97.81]	96.91	99.43	98.16

TL = transfer learning; KD = knowledge distillation; Freeze = layer freezing.

Moving on to the results obtained by training the model on all four magnifications sequentially. First, we check the results with the addition of just transfer learning (TL), which results in the average accuracy of the model lowering from around 98% to slightly above 93%. A similar conclusion can be drawn from the results of just knowledge distillation (KD), where the average falls to just below 91%. The usage of both of these, along with the model, results in the highest average that we have been able to achieve of around 95.43%.

We have also trained a model on the entire BreakHis dataset with all the magnifications combined for one train and one test set. This does not include successive training and does not involve TL or KD. The results of this model show how the level of improvement that can be achieved by the proposed model, with the addition of successive magnification training with TL + KD. While the 40× magnification is relatively close in accuracy to the proposed model (1% difference), the later magnifications (especially 400x) show a larger gap emerging between the two models in terms of results.

Another comparison that needs to be made here is between the various forms of the MobileVit versions, namely S, XS, and XXS. As previously mentioned, the paper chose the XS version of the model as it provides the best balance of performance-to-accuracy. When comparing to the smaller XXS model, the accuracy drop, especially in the earlier magnifications, makes it not suitable for this paper. When comparing to the large S model, while the accuracies of the later magnifications are comparable and sometimes slightly higher than the chosen model, this gain is offset by the much larger drop in accuracy in earlier magnifications, along with its increased performance requirement. To strengthen the reliability of these results, additional variability analysis was done to account for differences and variations between multiple training runs. Each experiment was repeated 3 times, and the mean and SD of the evaluation metrics was calculated and observed to be consistently low (within ± 0.5–1.0%). This analysis indicates stable model performance.

**Fig. 4:**
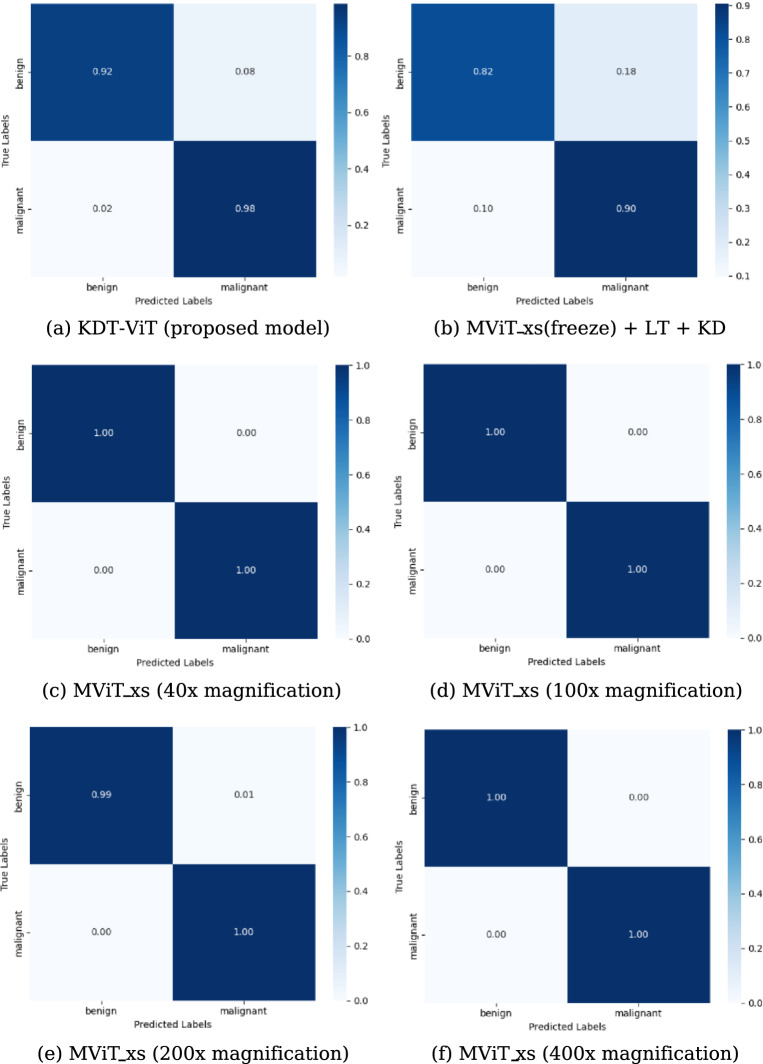

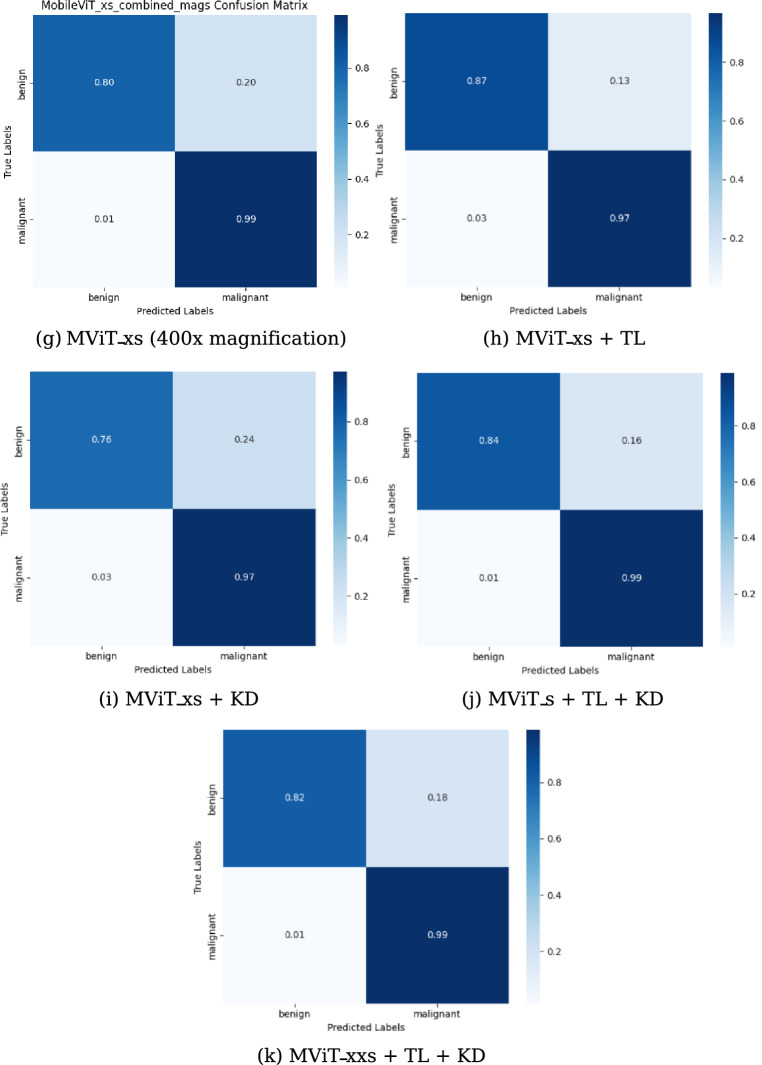
Confusion Matrices for training of MViT on BreakHis.

**Fig. 5:**
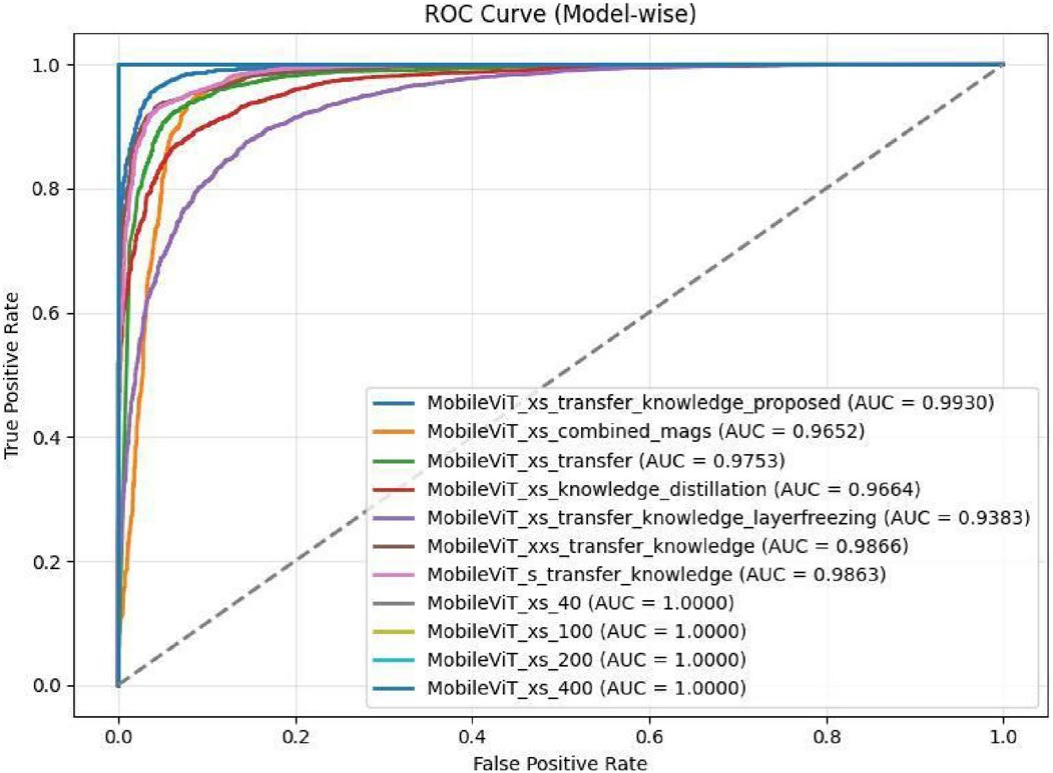
ROC curve results for training of MViT on BreakHis.

The results are further supported by confusion matrices for each training and by ROC curve results. The confusion matrix, depicted in Fig. 4, is common for classification tasks. The matrix is configured to highlight the four major characteristics in a confusion matrix: the true positive, true negative, false positive, and false negative values. The receiver Operating Characteristic (ROC) curve depicted in Fig. 5 is another common method for evaluation when it comes to classification models. The ROC curve works on the principle of the trade-off that occurs between the true positive rate and the false positive rate at multiple threshold settings. Here, all of the separate magnification trainings get a 1.0 AUC, while the proposed model achieves a very close 0.993 AUC, with other versions being lower than this. A tabular form has been given for all of the ROC-AUC values values in Table 2.

**Table 2:** ROC-AUC comparison across model configurations.

Model configuration	ROC-AUC
MobileViT_xs (40×)	1.0000
MobileViT_xs (100×)	1.0000
MobileViT_xs (200×)	1.0000
MobileViT_xs (400×)	1.0000
MobileViT_xs (all magnifications combined)	0.9652
MobileViT_xs + Transfer Learning (TL)	0.9753
MobileViT_xs + Knowledge Distillation (KD)	0.9664
MobileViT_xs + TL + KD + Layer Freezing	0.9383
MobileViT_s + TL + KD	0.9863
MobileViT_xxs + TL + KD	0.9866
**KDT-ViT (Proposed)**	**0.9930**

Bold is used to differentiate the proposed model results from the others present in the table.

While the confusion matrices and ROC curves show strong overall performance, class-level analysis reveals that the model achieves high sensitivity for malignant cases with few false negatives, while some benign samples are misclassified as malignant. Errors mostly occur in low-contrast images, especially at higher magnifications, indicating challenges in fine-grained texture differentiation.

Although confusion matrices provide a quantitative overview of performance, detailed analysis of misclassified samples reveals several recurring patterns. Errors were frequently observed in images exhibiting low contrast, overlapping cellular structures, or ambiguous morphological characteristics where benign and malignant regions share similar textures. These findings suggest that fine-grained cellular boundaries remain challenging even for transformer-based models. A more comprehensive pathology-guided error analysis will be explored in future work.

## Discussion

Here, we present the comparisons of the results obtained above with other models that have been used on the same dataset that use similar techniques. We conduct ablation studies to evaluate how effective each of the processes, like transfer learning and knowledge distillation, was on its own as compared to the proposed model. Additionally, to improve model interpretability and to qualitatively verify the spatial focus of the proposed model, Gradient-Weighted Class Activation Mapping (Grad-CAM)^17^ was applied to the proposed KDT-ViT model.

The observed performance trends across magnification levels can be interpreted in the context of underlying histopathology characteristics. Lower magnifications primarily capture global tissue architecture and structural organization, whereas higher magnifications reveal cellular morphology and nuclear details that are critical for distinguishing benign and malignant patterns. The improved performance at higher magnifications suggests that the model benefits from access to fine-grained cellular information, while performance variations at lower magnifications may be associated with reduced visibility of discriminative microstructural features. These findings indicate that the proposed cross-magnification learning strategy helps the model leverage complementary information present at different histopathology scales.

### Model interpretability with Grad-CAM

**Fig. 6:**
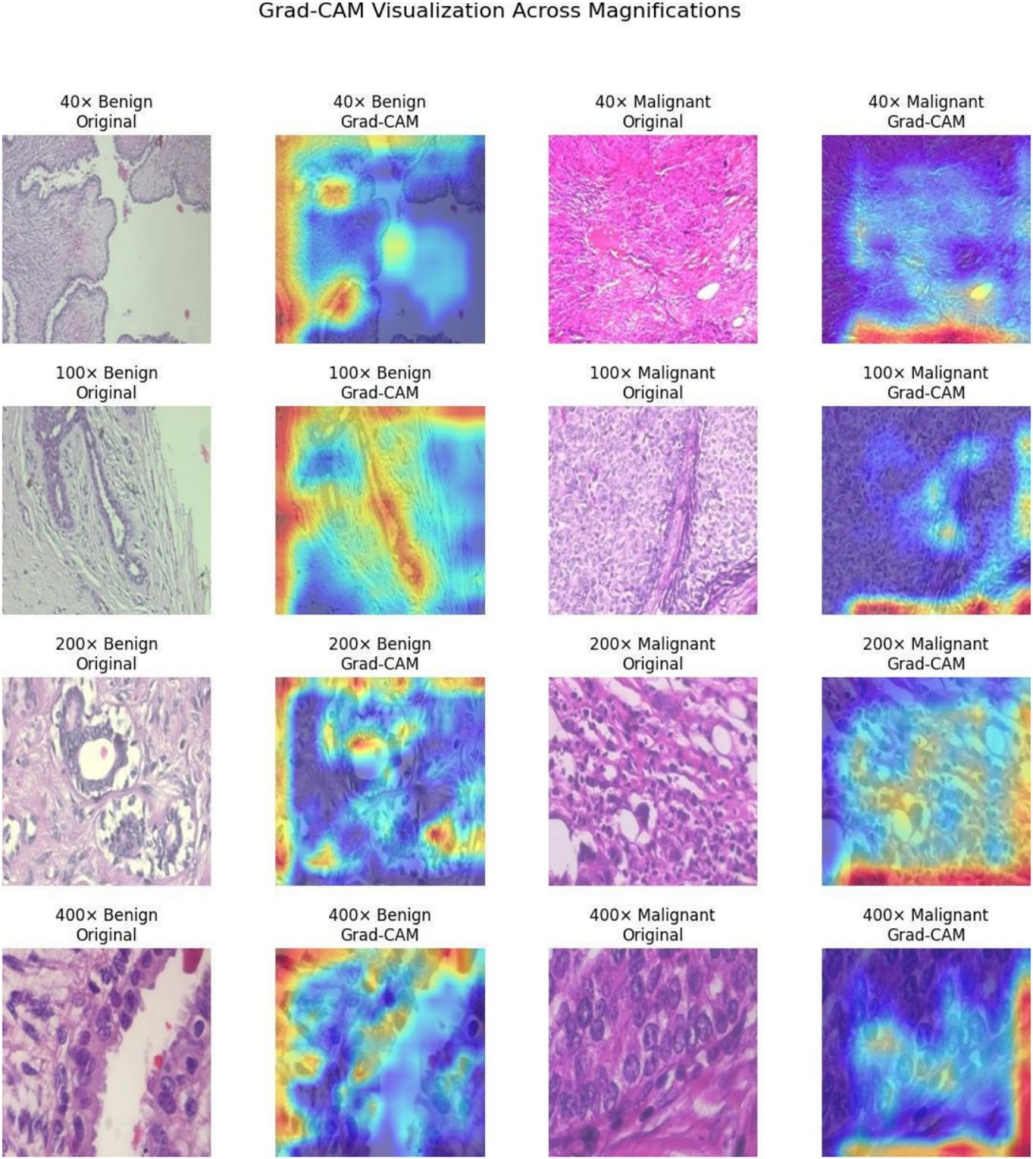
Grad-CAM at each magnification.

One of the criticisms levied against transformer models is their lack of interpretability. For this reason, we have chosen to apply a Grad-CAM^17^ on the proposed model to show and verify its spatial focus. Grad-CAM was used to generate a heatmap as an overlay on the images in the BreakHis dataset. Two images were chosen at random from each magnification, one benign and the other malignant. These heatmaps demonstrate which parts of the photos most contributed to the benign and malignant predictions made by the model, where the warmer regions correspond to higher model attention, while the cooler regions demonstrate lower model attention. It highlights the image regions that influenced the model’s decision-making the strongest. The Grad-CAM maps were generated from the second last layer of the proposed KDT-ViT model as this layer proves ideal in preserving the balance between sufficient spatial information and high-level contextual features. The images with their corresponding heatmaps are shown in the Fig. 6 below.

Although Grad-CAM visualizations provide qualitative insight into the regions influencing model predictions, quantitative validation of attention alignment was not performed in this study. Such evaluation typically requires pixel-level lesion annotations or expert-defined regions of interest, which are not available for the BreakHis and BACH datasets. Consequently, Grad-CAM is used here as a qualitative interpretability mechanism, while future work will explore quantitative overlap-based evaluation using pathologist-annotated datasets.

While Grad-CAM visualizations presented in this work primarily illustrate correctly classified samples, additional failure cases were analyzed during evaluation to better understand model limitations. Misclassified examples commonly exhibited diffuse or fragmented attention regions, often corresponding to ambiguous tissue morphology, low contrast structures, or visually similar benign and malignant patterns. Due to space constraints, these additional visualizations are not included in the manuscript; however, they highlight important directions for future interpretability studies involving systematic analysis of failure cases.

### Comparisons with the state of the art

Two major forms of comparison are presented in this section. First, the proposed MobileViT-based model (XS variant) is compared with other variants of the Mobile-ViT architecture. Second, comparisons are made against Vision Transformer and convolutional neural network approaches reported in prior literature. Beyond numerical performance differences, emphasis is placed on the methodological limitations of existing approaches when applied to cross-magnification histopathology tasks along with computational efficiency, parameter count, and multi-magnification capability.

The Fuzzy-ViT model proposed by Qiankun Li et al.^7^ reports results only for the 200× magnification setting. While strong performance is achieved, the lack of evaluation across multiple magnifications limits its applicability to realistic clinical scenarios where magnification varies. In comparison, the proposed model maintains consistent performance across magnification levels and achieves improvements of 1.61% in accuracy, 1.14% in precision, 7.35% in recall, and 4.2% in F1-score at 200x.

Another relevant approach is the multi-view augmented contrastive learning framework with knowledge distillation proposed by Si et al.^10^. Although this method leverages advanced augmentation strategies, it relies on multi-view processing pipelines that increase computational complexity and training overhead. In contrast, the proposed approach uses progressive magnification transfer with a lightweight architecture, achieving approximately 8–10% higher accuracy across magnifications while maintaining lower computational requirements.

The ST-Double Net proposed by Shengnan Hao et al.^8^, based on a two-stage Swin Transformer architecture, demonstrates strong feature extraction capability but introduces higher parameter count, higher model complexity, and reduced suitability for lightweight deployment. In contrast, the proposed model improves accuracy by approximately 3% at both the 200× and 400× magnification levels while preserving efficiency suitable for edge or resource-constrained applications.

Overall, many existing works train independent models for each magnification or rely on computationally heavy architectures. This limits their scalability and practical deployment in real-world clinical workflows where magnification variability is common. The proposed KDT-ViT framework addresses this gap by enabling efficient progressive cross-magnification learning within a single lightweight,low-parameter model, making it more practical for real-world clinical deployment. Additional numerical comparisons are provided in Table 3.

**Table 3:** Performance comparison with state of the art literature.

Model	Dataset	Mag	Acc (%)	Pre (%)	Rec (%)	F1 (%)
Fuzzy ViT^7^	BreakHis	200×	94.96	94.32	92.43	93.37
CPKD^10^	BreakHis	40×	83.99	99	96.01	97.85
		100×	86.01	98.25	97.02	97.63
		200×	90.63	99.68	98.74	99.21
		400×	89.40	96.65	97.12	96.88
Swin^8^	BreakHis	40×	95.95	95.99	95.75	95.78
		100×	93.72	93.65	91.69	92.46
		200×	93.00	90.41	91.33	90.82
		400×	92.52	94.50	89.75	91.38
ResNet-18 + attention^6^	BreakHis	40×	94.13	75.39	68.83	69.70
		100×	92.71	68.02	54.92	59.64
		200×	92.40	65.42	59.33	61.71
		400×	90.79	58.50	49.13	51.86
RAnet + Data Balance + ADSVM^9^	BreakHis	40×	94.43	93	94.21	93.56
		100×	98.31	98.12	97.8	98.02
		200×	99.14	98.62	99.05	98.93
		400×	93.35	92.27	92.76	92.45
VGGIN-Net^5^	BreakHis	40×	92.39	–	–	95
		100×	91.34	–	–	94
		200×	91.55	–	–	94
		400×	88.52	–	–	92
SwinCNN^18^	BACH	200×	92.89	93	91.4	93
Ensemble model^11^	BACH	200×	92	92.16	91.94	92.15
KDT-ViT (proposed)	BACH	200×	98.00	96.60	99.50	98.03
KDT-ViT (proposed)	BreakHis	40×	92.58	91.56	98.25	94.79
		100×	95.10	93.89	99.37	96.55
		200×	96.57	95.46	99.78	97.57
		400×	97.47	96.91	99.43	98.16

Each of the works cited here works on different principles for training and may have differences in terms of data splitting, augmentation, magnification handling, etc. This means that a direct comparison between results from the models should not be used as an indicator of a superior model or training strategy. Instead, this comparison with the state of the art indicates that the training strategy used in this paper is comparable to the findings of other works on similar datasets and training methods.

The main method to understand how the proposed model and its training methods improve the training of MViT on these datasets is by comparing them in the same pipeline. This is what has been detailed in the next section.

### Ablation studies

Ablation studies were conducted to evaluate the individual impact of key components, such as transfer learning and knowledge distillation, on the final model performance. This involved re-training the model while selectively excluding these techniques to observe the resulting changes in accuracy.

#### Model performance without knowledge distillation

In this section, the proposed model is retrained without the use of KD. TL is still used on the model. This gives the details on how each of these methods improves or hurts the performance of the base mobile ViT model. The results show that without the addition of knowledge distillation, while the model could not be considered to be severely affected by chronic forgetting, it has definitely taken a hit in terms of accuracy especially in earlier magnifications which also affects the overall average accuracy and is against the stated goal of closing the gap between the training on all magnifications combined with training individually on each magnification. Hence, the outcome of this ablation study shows that the removal of knowledge distillation has a substantial effect on the results that we obtain as compared to the proposed KDT-ViT model, since no teacher model existed to help guide the student model. Table 4 showing these results is presented below.

**Table 4:** Performance Comparison without knowledge distillation.

Metrics	Magnification	MViT_xs + TL (no KD)	KDT-ViT (proposed)
Accuracy (%)	40×	87	92.58
	100×	91	95.10
	200×	95	96.57
	400×	97	97.47
Precision (%)	40×	86	91.56
	100×	92	93.89
	200×	95	95.46
	400×	97	96.91
Recall (%)	40×	96	98.25
	100×	94	99.37
	200×	98	99.78
	400×	99	99.43
F1 score (%)	40×	91	94.79
	100×	93	96.55
	200×	97	97.57
	400×	97	98.16

#### Model performance without transfer learning

This ablation study was performed by using KD without TL. This means that while teacher and student models do exist, every student model is a new, previously untrained model instead of already being trained on earlier magnifications. This form of setup can pose the risk of chronic forgetting, which is when the model, during training on multiple different sets of data in different sessions without transferring its knowledge using TL, forgets details relating to previous data points. This can also be seen here, as while the accuracy of higher magnifications (200× and 400×) changes by around 0–1%, the accuracy of the 40× magnification drops by a very substantial 12%, which signals that the model has started to forget details on earlier magnifications. Table 5 showing these results is presented below. Hence, the outcome of this ablation study shows that the results we obtain by removing transfer learning, as compared to the proposed KDT-ViT model, show a case of chronic forgetting, which means that the model has forgotten a lot about older magnifications which means that the model would not perform well in a real-world scenario on smaller magnifications.

**Table 5:** Performance Comparison without transfer learning.

Metrics	Magnification	MViT_xs + KD (no TL)	KDT-ViT (proposed)
Accuracy (%)	40×	80	92.58
	100×	87	95.10
	200×	93	96.57
	400×	96	97.47
Precision (%)	40×	80	91.56
	100×	87	93.89
	200×	94	95.46
	400×	97	96.91
Recall (%)	40×	93	98.25
	100×	95	99.37
	200×	97	99.78
	400×	99	99.43
F1 score (%)	40×	86	94.79
	100×	91	96.55
	200×	95	97.57
	400×	98	98.16

#### Model performance with additional layer freezing

In this study, the models were trained like the proposed model, but freezing of the first 30% of the layers for every model before the start of the next training session on the next magnification was used. This choice was motivated by the observation that early convolutional layers capture generic low-level features (e.g., edges and textures) that are largely invariant across magnifications, while deeper layers encode task-specific representations. Freezing a smaller portion provided limited regularization benefit, whereas freezing too many layers restricted adaptation to magnification-specific patterns. The 30% configuration provided a practical balance between feature reuse and model flexibility. This means that before training on 100×, 200×, and 400× datasets, the model had its initial 30% of layers frozen while ensuring a similar reduction in total number of unfrozen parameters. The results for this are given below. The results of this ablation study show that, as compared to the proposed KDT-ViT model, the addition of freezing the lower 30% of the layers of the model after training on each magnification does not improve results from the model for older magnifications; instead, it actually hinders the model’s ability to learn newer magnifications. This shows that there is a balancing act involved, where prioritizing the older magnifications can lead to better results; however, going too far in prioritizing the older magnifications can hinder the model’s performance and prevent the model from training and understanding later magnifications. Hence, the addition of KD and TL causes the overall average accuracy to increase as the model reaches a good equilibrium between former and later magnifications; however addition of freezing layers tipped the balance between the magnifications, hindering the overall performance of the model on all magnifications. Table 6 showing these results is presented below.

**Table 6:** Performance comparison with layer freezing.

Metrics	Magnification	MviT_xs Freeze + TL + KD	KDT-ViT (proposed)
Accuracy (%)	40×	85	92.58
	100×	85	95.10
	200×	86	96.57
	400×	89	97.47
Precision (%)	40×	89	91.56
	100×	94	93.89
	200×	90	95.46
	400×	89	96.91
Recall (%)	40×	89	98.25
	100×	85	99.37
	200×	90	99.78
	400×	96	99.43
F1 score (%)	40×	89	94.79
	100×	89	96.55
	200×	90	97.57
	400×	92	98.16

### Clinical workflow integration

In a clinical setting, the proposed model can function as a tool used by medical professionals for decision support rather than a replacement of the medical expert/ pathologist. The KDTViT model can be integrated into the workflow after scanning the slides, where the images in the slides are sent into the system for analysis. The system provides class predictions along with a confidence interval and a visual explanation (Grad-CAM) that can help pathologists reach a conclusion or prioritize suspicious regions for detailed examination.

### Limitations

A potential limitation of this work is the presence of staining variability and scanner-related differences inherent to histopathology datasets. Variations in staining protocols, illumination conditions, and slide acquisition devices may introduce color and texture shifts that can influence model generalization. Although basic normalization and data augmentation were employed to improve robustness, such approaches may not fully eliminate domain-specific bias. Consequently, performance may vary when applied to data acquired from different institutions or scanners.

Additionally, the dataset may exhibit sampling bias, including imbalances in tissue types, magnification distributions, or patient representation, which can influence model performance. While normalization and data augmentation were applied, these techniques may not fully eliminate such biases. Additionally, sampling bias may be exhibited by the dataset, including imbalances in magnification distribution, patient representation, or tissue types. This can influence the performance of the model. The implementation of normalization and data augmentation, though helpful, does not fully eliminate these biases.

Along with this, the lack of external validation on independent similar datasets restricts the assessment of real-world generalization across institutions.

Future work will investigate these points to further improve robustness and reduce domain-related bias.

## Conclusion

This work presents a unified framework for breast cancer classification in histopathology images using a lightweight MobileViT-based architecture enhanced with transfer learning and knowledge distillation. Experimental results demonstrate that the proposed approach achieves strong and consistent performance across multiple magnification levels, reducing the need for magnification-specific models commonly used in prior work.

Beyond performance improvements, the proposed strategy highlights the effectiveness of combining cross-magnification learning with efficient transformer architectures for medical imaging tasks. The resulting model offers practical advantages for real-world deployment by simplifying the training pipeline and improving robustness to magnification variability.

Future work will focus on improving generalization across diverse staining conditions, conducting external validation on multi-center datasets, and expanding interpretability analysis to further enhance clinical reliability.

## Data Availability

The datasets used and analysed during the current study are publicly available from their original sources. The BreakHis dataset is publicly available at^2^, link https://web.inf.ufpr.br/vri/databases/breast-cancer-histopathological-database-breakhis/. The BACH dataset is available at^15^, link https://iciar2018-challenge.grand-challenge.org/Dataset/.
